# A Flavonoid on the Brain: Quercetin as a Potential Therapeutic Agent in Central Nervous System Disorders

**DOI:** 10.3390/life12040591

**Published:** 2022-04-15

**Authors:** Dagmara Wróbel-Biedrawa, Karolina Grabowska, Agnieszka Galanty, Danuta Sobolewska, Irma Podolak

**Affiliations:** Department of Pharmacognosy, Faculty of Pharmacy, Medical College, Jagiellonian University, Medyczna 9, 30-688 Krakow, Poland; dagmara.wrobel-biedrawa@uj.edu.pl (D.W.-B.); agnieszka.galanty@uj.edu.pl (A.G.); danuta.sobolewska@uj.edu.pl (D.S.); irma.podolak@uj.edu.pl (I.P.)

**Keywords:** quercetin, CNS, cognition, neuroinflammation, antioxidant, neurodegeneration, nanoformulations

## Abstract

Quercetin is one of the most common, naturally occurring flavonoids, structurally classified to the flavonol subfamily. This compound, found in many edible and medicinal plants either as a free or glycosidated form, has been scientifically exploited for many years, and one could hardly expect it could be a hero of some additional story. Commonly recognized as an anti-inflammatory agent, quercetin not only limits capillary vessel permeability by inhibiting hyaluronidase but also blocks cyclooxygenases and lipoxygenases. As a typical flavonoid, it is also known for its antioxidant effect, which was confirmed by many in vitro and in vivo studies. Throughout the years, numerous other activities were reported for quercetin, including antidiabetic, anti-proliferative, or anti-viral. Of note, recent data have revealed its potential role as a therapeutic agent for several central nervous system disorders. This review provides an overview of available experimental data on quercetin and its complexes with respect to central nervous system diseases, with a main focus on some aspects that were not discussed previously, such as anti-anxiolytic effects, anti-Huntington’s disease activity, or therapeutic potential in brain cancer. Moreover, quercetin’s protective role in some of these diseases is discussed, especially as an anti-neuroinflammatory agent. Bearing in mind the poor bioavailability of this compound, possible options that would enhance its delivery to the site of action are also presented.

## 1. Introduction

**Figure 1:**
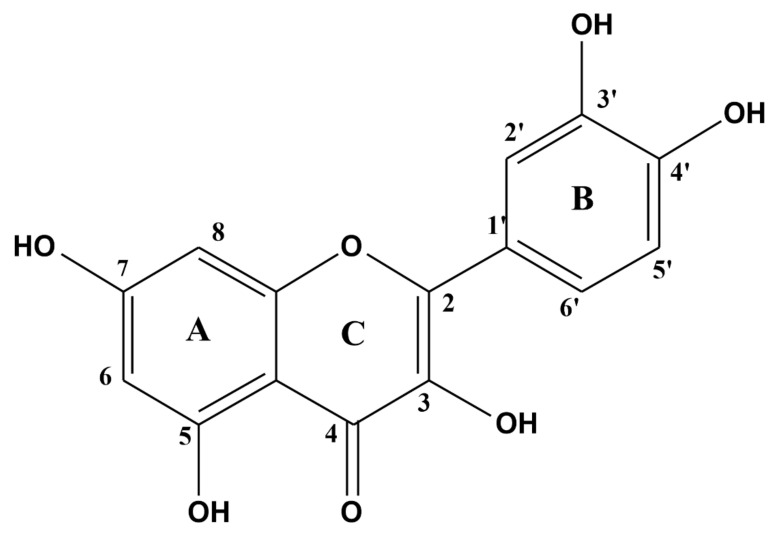
Structure of quercetin.

**Figure 2:**
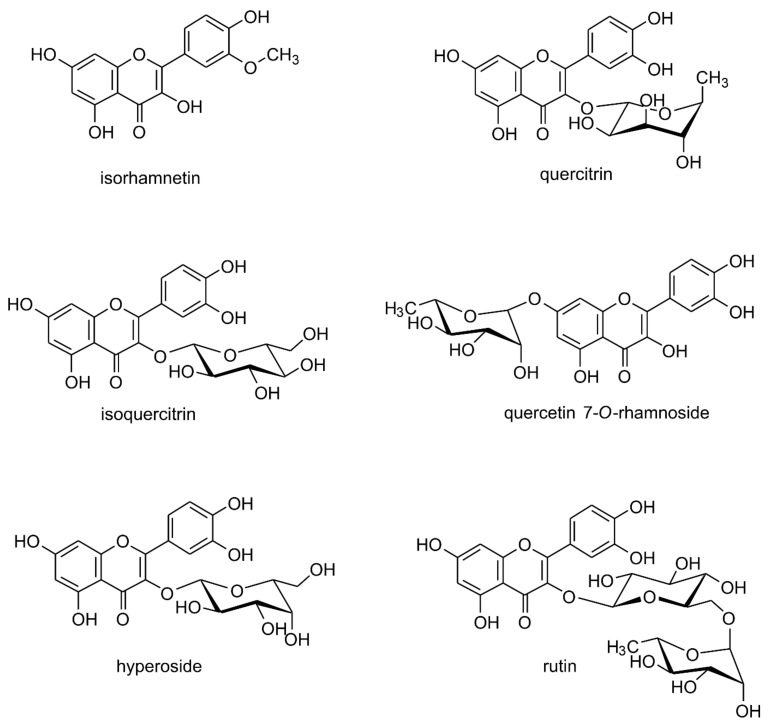
Naturally occurring quercetin derivatives: isorhamnetin (3-methyl ether of quercetin) quercitrin (quercetin 3-*O*-rhamnoside), isoquercitrin (quercetin 3-*O*-glucoside), quercetin 7-*O*-rhamnoside, hyperoside (quercetin 3-*O*-galactoside), rutin/rutoside (quercetin 3-*O*-rhamnozyl-(1 → 6)-glucoside).

Quercetin (Que) is a flavonol-type flavonoid, which was isolated for the first time in 1854 from its glycoside, quercitrine (Figure 1 and Figure 2) [1]. It is found commonly in herbaceous and woody plants, especially in glycosylated forms (Figure 2) [2,3]. Chemically, it derives from diphenylpropane skeleton (C6–C3–C6) and contains a heterocyclic ring (C) with an incorporated oxygen atom (pyran) (Figure 1).

The protective and disease-preventive activity of quercetin as a food ingredient is well-known. A great number of studies confirmed its anti-inflammatory and antioxidant effect [2,3,4,5]. Furthermore, cytotoxic, anti-proliferative, antidiabetic, anti-obesity, and anti-microbial activities were reported [1,3,4]. Recent data indicate that quercetin may also play a beneficial role in central nervous system (CNS) disorders [1,6,7,8,9]. The idea that Que may be of use as a potential therapeutic agent is based on its well-known antioxidant (recognized as one of the most potent antioxidant flavonoids, considered to be stronger than vitamin C or tocopherols [10]) and anti-inflammatory efficacy, which contribute to neuroprotective activity. It is generally accepted that the central nervous system, especially the brain, is a specific environment, characterized by a high level of easily peroxidizable unsaturated fatty acids and iron ions with pro-oxidative potential. Taken together, along with the relatively low level of antioxidant protection (superoxide dismutase (SOD), glutathione (GSH) transferase (GST), metal-binding proteins, H_2_O_2_-removing enzymes), this makes the brain especially vulnerable to the destructive action of reactive oxygen species (ROS), leading to cell degeneration or even death [11]. Provided the brain consumes glucose as its main energy source, a high amount of oxygen (20% of overall organism use) is needed for the mitochondrial metabolism. As a result, ROS, which are byproducts of the electron transport chain, are generated at a relatively high level, as compared to other organs. Oxidative stress (imbalance between ROS and/or reactive nitrogen species (RNS) and defense antioxidant system) may produce oxidative damage of cellular macromolecules and subsequent metabolism impairment, which leads to death (apoptosis) of neurons and glial cells [12]. Furthermore, the role of inflammation in the pathogenesis of various CNS diseases (neurodegenerative disorders, schizophrenia, depression, cancer) has been recently proven by several research groups [13,14]. Neuroinflammation can result from some primary disadvantageous alterations specific to some disorders, e.g., it can be induced by β-amyloid aggregates or hyperproliferated cells of a neoplasm. Moreover, neurons are not the only ones that can be affected by the inflammation process. Gliosis (e.g., astrocytosis) is often involved in pathological changes related to neurodegeneration and other CNS disorders [13,14].

A vast amount of experimental data has been accumulated in recent years, suggesting that Que could be a promising molecule for further development as a therapeutic agent. Moreover, it is recognized as safe and, in recommended doses, is deprived of any side effects.

Several reviews published recently have dealt with the various biological activities of Que [1,2,3,9,15,16,17]. Especially widely reviewed is its neuroprotective activity, with an insight into mechanistic aspects, as well as its potential role in Alzheimer’s disease, cancer, or other neurological disorders [6,18,19,20,21,22,23,24,25]. Furthermore, some reports focused on potential molecular/cellular targets in cognitive impairments [26,27] and others on Que’s efficacy in animal models of AD [28]. Recently, Silvestro et al. have reviewed the anti-depressive-like potential of Que in acute and chronic stress-induced animal models of depression [7]. A single review paper was devoted to Que’s role in Parkinson’s disease, with a main focus on its molecular and cellular aspects [29]. Nevertheless, to date, no review has provided an overview of different Que activities with regard to all the most relevant CNS disorders.

Therefore, the aim of the current review was to critically verify the perspective of Que use in CNS diseases, with special attention paid to aspects that have not been mentioned in any of the previous reviews, such as anti-anxiolytic, anti-Huntington’s, and anti-Parkinson’s disease preclinical activity. Taking into account the severity and treatment failures of brain cancer, the potential anti-tumor role of Que is discussed as well. Furthermore, an important issue discussed in this review with regard to CNS studies on Que relates to novel formulations, significantly enhancing efficacy of this compound. These are essential for further development of Que in terms of clinical applications in CNS disorders.

### Methods

Scopus, Medline, and PubMed databases have been searched through with the following key words inquiries: “quercetin” +: “central nervous system”, “depression”, “neurodegenerative”, “mental”, “brain cancer”, “dementia”, “Parkinson’s disease”, “Huntington’s disease”, “anxiety”, “novel delivery system”, “novel formulations”. A literature search was conducted until 26 February 2022. About 43,000 articles concerning quercetin and its novel formulations in terms of activity in CNS have been found. For the purpose of this review, only full-text articles in English from years the 2012–2022, with regard to some important publications from earlier years, have been chosen. Finally, 145 articles are referenced to in this paper.

## 2. Metabolism and Bioavailability of Quercetin

The high lipophilicity of quercetin (Que) determines its poor water solubility and subsequent bioavailability after ingestion. A limited percentage of ingested Que that reaches circulation results in small concentrations available for peripheral tissues and organs. The absolute bioavailability from aqueous solution was estimated at 16% in rats, whereas, for ethanol-PEG, it reached 27.5% [30,31]. Most dietary Que is in the form of glycosides (Figure 2). In gastro-intestinal tract glycosides, before their absorption takes place, they are hydrolyzed by β-glucosidases to the aglycon. Then, they are converted into glucuronide and sulphate conjugates or methylated derivatives. These are the forms of Que present in circulation. Interestingly, an absorption to circulation of quercetin from some glucosides (quercetin 3-*O*-glucoside, i.e., isoquercitrin, the main form of quercetin found in onion, *Allium cepa*) is greater than in the case of unglycosylated, pure Que or its other glycosides, e.g., rutinoside (rutin) or galactoside (found in apples) [30]. This can be a result of the presence of glucose intestinal carriers (sodium-dependent glucose transporter 1, SGLT-1) which can actively transport some glucosides through the membrane of enterocytes. Ishisaka et al. [32] showed that Que metabolites crossed the blood–brain barrier (BBB) and reached high enough amounts in the brain tissue to exert its pharmacological activity. However, in many in vivo investigations of Que activity in CNS disorders, effective doses were relatively high. A characteristic feature of substances with low bioavailability is that to achieve pharmacological effect, high doses of the drug are needed. To improve systemic bioavailability, various methods that increase solubility are employed. Among them, particle size reduction (nanoparticles, nanosuspensions) and chemical modification (derivatization, complexation) can be mentioned.

## 3. Quercetin-Loaded Nanocarriers—New Delivery to Better Availability

**Figure 3:**
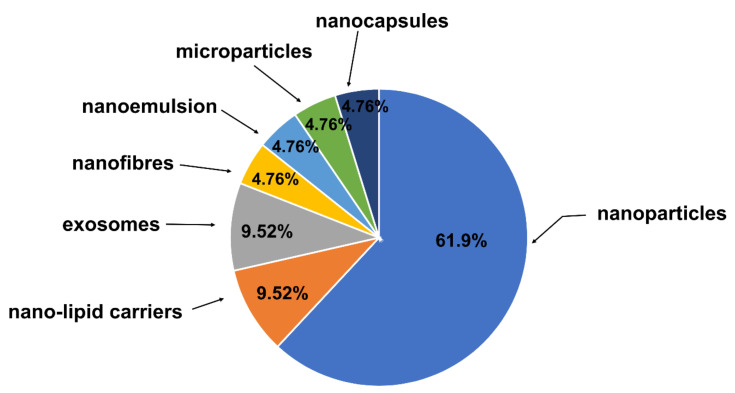
Percentage of novel formulations of quercetin investigated in vitro and in vivo in terms of neurodegenerative disorders; results published in 2012 to 2022.

To improve the biodistribution profile of Que, different formulations have been developed recently (Table 1). The most popular of the analyzed forms are quercetin-loaded nanoparticles (Figure 3) [33]. Nanoparticles allow an increase in the bioavailability of Que and overcome limitations such as its solubility and dissolution profile, and digestive enzyme activity in the gastrointestinal tract. The essential parameters for the increased effectiveness as well as penetration of the blood–brain barrier are the size and weight of the nanoparticles. It seems that that the range of 20 to 100 nm is the optimum size.

One of the formulations extensively studied which can carry the loaded drug to the desired target area using an external magnetic field are Fe_3_O_4_ nanoparticles (SPIONs) [40,47].

In vivo studies demonstrated that the concentration of Que in the brain, when delivered in conjugates with superparamagnetic iron oxide nanoparticles (QT-SPION; 30–50 nm in diameter; at a dose of 50 and 100 mg/kg/d), was about 7- and 10-folds higher in comparison to free Que.

Among the different nanocarriers analyzed, nanostructured lipid carriers are considered one of the most promising therapeutic Que delivery systems, providing stable and effective formulation design. Kumar et al. [36] investigated naturally derived and semi-synthesized lipid composition involving phospholipids, vitamin E acetate, and glyceryl behanate. Quercetin-loaded nanolipidic carriers (NLCs; employing phospholipids and tocopherol acetate) and quercetin-loaded solid lipid nanoparticles (SLNs), prepared by a simple emulsification technique, enhanced the brain delivery of Que by 5.6 and 3.2 times, respectively. However, quercetin-loaded, cationic, nanostructured lipid carriers (QR-CNLC) failed to accumulate higher Que content in the brain tissue than Que suspension, probably due to too big particle size (126.6 ± 8.48 nm) [34].

Drug delivery using biodegradable, biocompatible, non-toxic carriers has focused great attention in recent years. One of interesting example that has been extensively investigated is poly(n-butylcyanoacrylate) (PBCA). Quercetin-loaded poly(n-butylcyano acrylate) nanoparticles (QT-PBCA NPs) and QT-PBCA NPs coated with polysorbate-80 (P-80) (QT-PBCA + P-80) enhanced Que bioavailability by 2.38- and 4.93-fold, respectively, as compared to free Que. PBCA NPs coated with P-80 improved the oral bioavailability of Que by enhancing its transportation ability to the brain [35]. Exosomes are vesicles of nano-scale size produced by living cells. They have been recently recognized as beneficial drug carriers, as they have some advantages in comparison with other drug delivery systems, including good biocompatibility, low immunogenicity, and high level of transmission. As exosomes are naturally targeted to some regions, they need to be specifically modified so that they achieve mostly the CNS cells. Moreover, a pharmacokinetic study in rats showed that after i.v. injection of the same amount of Que given alone or in an exosome delivery system, better pharmacokinetic parameters were revealed in the latter case (half-life: 89.14 min vs. 36.47 min, maximum plasma concentration: C_max_, 2.5 mg/L vs. 1.31 mg/L) [37].

Another promising drug delivery system for this flavonoid involves selenium–quercetin nanoparticles, which are used to form a composite with an increased aqueous solubility and, consequently, better bioavailability [48]. The study assessing its efficacy was performed in vitro, but interestingly, it was found that the composite could inhibit the formation of Aβ fibrils as well as revealed antioxidant and protective activity from H_2_O_2_ damage towards the PC12 cell line.

## 4. Quercetin in Neurodegenerative Diseases—Dementia and Alzheimer’s Disease, Parkinson’s, Huntington’s Diseases

Neurodegeneration refers to any pathological state characterized by neuronal damage, impeding neuronal functionality, or neuronal death. Neurodegenerative diseases are neurological disorders in which specific groups of neurons (in terms of function and anatomic localization) are affected. From among a great number of neurodegenerative disorders, only a few have gained wider interest as research objects for studying potential therapeutic role of quercetin, that is, Alzheimer’s disease (AD), Parkinson’s disease (PD), Huntington’s disease (HD), and amyotrophic lateral sclerosis (ALS).

### 4.1. Dementia and Alzheimer’s Disease

Dementia is the disorder of cognitive functions such as remembering, thinking, reasoning, and association, most commonly resulting from the degeneration of cerebral cortex neurons. Apart from memory loss, other manifestations of this disease include executive dysfunction and behavioral alteration. The progressive nature of the impairment eventually makes independent existence of an individual impossible. The most representative example of dementia is AD (with a prevalence of 60–70% [49]), but it should be remembered that cognitive disturbances can accompany as a co-symptom at least 50 other diseases and not all of them are of neurodegenerative origin. These include metabolic, toxic, infectious, ischemic, or traumatic changes, as well as mental and behavioral disorders (depression, schizophrenia, severe anxiety disturbances).

As was mentioned earlier, several reviews discussed quercetin’s (Que) effectiveness in cognitive decline with proposed molecular mechanisms responsible for its activity. In recent years, there have been some additional studies confirming the pro-cognitive activity of Que in AD [50,51]. In order to present that this effect goes far beyond antioxidative and anti-inflammatory activity of Que and is in part specific towards alterations characteristic of neurodegenerative dementia and in part non-specific, several mechanisms previously reported [21,27] and regarded as the most important are briefly summarized below. These include cholinergic, pro-neurotrophic, antioxidant, and anti-inflammatory effects. A fairly novel approach to establish a possible anti-AD mechanism is insulin disruption in CNS (diabetes type 3).

#### 4.1.1. Cholinergic Effect

The earliest and most popular hypothesis which explains the pathogenesis and development of AD is the one based on cholinergic transmission deficiency. This involves damage of cholinergic neurons due to cell degeneration and, in consequence, a decline in the level of acetylcholine, which is eventually too low for a proper transmission in the brain regions responsible for cognition, i.e., the prefrontal cortex and hippocampus [52].

Que has revealed acetylcholinesterase (AChE) inhibitory activity, which was confirmed in several studies [53,54,55,56]. Noteworthy, this is the main mode of action of drugs currently used in the treatment protocols of dementia (mainly AD): donepezil, rivastigmine, and galantamine. However, their efficacy is recognized as low in improving cognitive decline, with no significant amelioration of behavioral symptoms [57,58]. On the other hand, Que, apart from its AChE-inhibitory activity, demonstrates a variety of additional effects (described below) which may be responsible for the overall wider end-effect of Que as compared to standard drugs.

#### 4.1.2. Pro-Neurotrophic Effect

A decline in the levels of neurotrophic factors is noted in some neurodegenerative and psychiatric diseases, e.g., dementia and depression. Increased expression of genes for these neurotrophic factors is responsible for neuronal plasticity, improving synaptic functions and synaptogenesis, neuronal survival, and promoting neurogenesis, the physiological processes that are essential for learning and memory and that are dysregulated or suppressed during neurodegenerative dementia [59].

Several studies showed stimulating effect of Que on brain-derived neurotrophic factors (BDNF) [60,61,62], nerve growth factor (NGF) activity, or cyclic-AMP-response element-binding protein (CREB) phosphorylation [20,63,64]. Provided that alterations leading to restoration of these processes last for days and weeks, it is not surprising that the effect of quercetin is observed after a longer period of the time [58,65,66,67,68,69].

#### 4.1.3. Anti-Inflammatory Effect

Inflammation is a hallmark of pathological alterations in AD. The well-known anti-inflammatory potential of Que was also confirmed in the context of CNS disorders in several studies conducted in the past decade [5,43,69]. Glial tissue plays an important role in the maintenance of CNS activity [70,71]. Astrocytes along with microglia regulate the release of cytokines (TNF-α, IL-1β) involved in neuroinflammation process, which is an immune response initiated by triggers such as protein aggregation or neuronal death. Suppressing inducible form of cyclooxygenase (COX-2) and different forms of lipoxygenase (LOX) reduces cytokines release.

Que suppressed activity of COX-2, LOXs [5]. Among many anti-inflammatory pathways, the inhibition of TNFα, NLRP3, Nf-κB, MAPK, and JNK was shown to be engaged in the pro-cognitive effect of Que [21,25,69]. Furthermore, antagonizing Toll-like receptors (TLR), which are essential in pro-inflammatory signals (cytokines, chemokines) produced by β-amyloid (Aβ), was also revealed [37]. Interestingly, it was noted that an anti-inflammatory effect can be achieved by the activation of enzymes belonging to sirtuin family (SIRT) or by enhancement of SIRT gene expression [69]. Finally, it was shown that Que is one of the flavonoids reducing the inflammatory profile of astrocytes exposed to IL-1β [72].

#### 4.1.4. Antioxidant Effect

Antioxidant activity is definitely the most profoundly investigated of all Que activities. As mentioned before, this ability is especially important in terms of neuroprotection. Oxidative stress is one of the factors responsible for harmful changes in the prefrontal cortex and hippocampal tissue, including cholinergic transmission [11].

Que neutralizes highly reactive oxygen/nitrogen species both directly, due to its chemical structure (see: Section 1 and Figure 1), or indirectly, by modulating antioxidative signaling pathways, such as the nuclear factor-like 2 (Nrf-2), regulating transcriptions of inducible anti-oxidant elements, paraoxonase-2 (PON2), a calcium-dependent lactonase with anti-oxidant functions, and the c-Jun N-terminal kinase (JNK3), participating in inflammatory, apoptotic processes, activated after exposure to, e.g., Aβ [21,73]. Que also affects several signaling molecules of the integrated stress response (ISR) (for the recent review-see [74]). The ISR can be assumed as a cellular restorative system of protein homeostasis. Que exhibited a potential to alleviate the level of mammalian activating transcription factor 4 (ATF4), an element of the ISR, increased in this model of rodent dementia [75]. Furthermore, a decrease in oxidative stress parameters were observed by reducing corticosterone level, which can suggest an involvement of quercetin in hypothalamus-pituitary-adrenal axis activity (HPA) [76]. Among many different effects exerted by Que, it is difficult, though, to assess to what extent the antioxidant activity influences the cognitive-enhancing response. Nevertheless, as it is known to participate in neuroprotective effects, its role in prophylaxis and slowing down the progression of degenerative/apoptotic changes within CNS can be assumed.

#### 4.1.5. Anti-Neurotoxic Protein Aggregates

An impact on neurotoxic protein aggregates, pathogenic structures observed in some of neurodegenerative diseases, was also described for Que. At the molecular level, the extracellular accumulation of Aβ aggregates (derived from amyloid precursor protein, APP) called senile plaques (SPs) and the hyperphosphorylation of tau proteins resulting in the formation of neurofibrillary tangles (NFTs) deposits and disorder of synaptic transmission are recognized as hallmarks of AD. SPs contain a vast number of inflammatory proteins enhancing the production of pro-inflammatory cytokines. Moreover, COX-2 enhances the activity of α-synuclein as well as γ-secretase (an enzyme engaged in Aβ formation), resulting in more Aβ being deposited.

As can be seen, the antioxidant and anti-inflammatory activities of Que may provide an additional beneficial effect in alleviating and delaying the progress of the disease. Furthermore, the mechanism of quercetin’s anti-Alzheimer’s dementia activity may be correlated with the direct suppression of Aβ formation and the stability of Aβ fibrils [77]. It was shown that the compound inhibited different steps of sclerotic plaques formation: suppressing β-secretase and aggregation of Aβ [21]. Moreover, a highly active cyclin-dependent kinase 5 (CDK5) stimulates tau hyperphosphorylation and further aggregation into NFTs. Of note, it was shown that CDK5 is a molecular target for some natural substances inhibiting hyperphosphorylated tau-induced neuronal pathology in mice [78]. Kuo [79] reported that Que is also effective in decreasing tau hyperphosphorylation.

Those recently widely studied therapeutic targets, such as Aβ aggregation, tau protein hyperphosphorylation, or the use of vaccines, seem to be promising; however, to date none of the clinical trials have succeeded.

#### 4.1.6. Improving Metabolic Disruption Effect

A cross-talk between metabolic factors and dementia seems to be obvious for researchers nowadays. It was shown, for example, that Aβ and insulin compete in biding to the insulin receptor [80]. Furthermore, insulin resistance and the impairment of leptin receptor function are linked to cognitive decline symptoms [81].

Interestingly, Que was proven to reverse cognitive deterioration, secondary to metabolic disturbances in animal models, although the effect seemed to be moderate or weak [82,83] Furthermore, Que preferentially exhibited pro-cognitive activity in Zucker rats lacking the leptin receptor but not in Wistar rats, with no defect in a gene for leptin receptor [84].

### 4.2. Parkinson’s Disease

The degeneration of basal ganglia neurons is often manifested by motor dysfunction. Hypokinetic disorder resulting from the dopaminergic nigrostriatal system damage, with symptoms such as bradykinesia, resting tremor, muscular rigidity, slowness of movements, and postural instability, are typical symptoms of Parkinson’s disease (PD). Additionally, nonmotor disruptions in automatic functions along with neuropsychiatric impairment (e.g., dementia) often/usually accompany the movement manifestations.

Unfortunately, to date, there are no effective methods that would inhibit neurodegenerative changes of dopaminergic neurons. Available pharmacotherapy is based on supplying dopamine precursors or on stimulation of dopamine synthesis in the remaining neurons. A couple of studies reported a beneficial effect of Que on motor dysfunctions in PD models. The administration of Que at a dose of 30 mg/kg in 6-hydroxydopamine (6-OHDA)-intoxicated rats for 14 consecutive days resulted in amelioration in the striatal dopamine and antioxidant enzyme levels in comparison with a PD-induced control. Additionally, there were fewer dead neurons observed in the Que-treated group. In another experiment, Que doses of 100 and 200 mg/kg, but not 50 mg/kg, given p.o. for 14 consecutive days alleviated significantly motor balance and coordination in the rotarod test in mice with 1-methyl-4-phenyl-1,2,3,6-tetrahydropyridine (MPTP)-induced PD [85].

Mu et al. [86] showed in a model involving unilateral lesion of striatum that the flavonoid suppressed dose-dependent tremor in rats but did not alter apomorphine-induced rotations; however the effective doses were very high (100, 200, 400 mg/kg). Furthermore, a reverse of the 5-HT depletion in the PD group was noted but with no change in the dopamine level. On the other hand, in a study utilizing a rotenone-induced PD in rats that confirmed movement improvement (catalepsy, rearing test, apomorphine test, grip strength test) after 50 mg/kg of Que was applied i.p., dopamine level, as well as oxidative balance, were restored [87]. What is more, in the above-mentioned experiment with MPTP-induced PD, Que at two higher doses improved the antioxidant enzymes (glutathione peroxidase, superoxide dismutase (SOD), Na^+^/K^+^-ATPase) supplies and elevated the levels of ACh and dopamine, whereas the levels of peroxidation product, 4-HNE, were reduced in the striatum [85]. Apparently, there is no consistency in results of the in vivo studies, especially in terms of dopamine-level regulation. Nevertheless, Que beneficial effect on motor deficits is noticeable, albeit the active doses were relatively high.

### 4.3. Huntington’s Disease

Basal ganglia disorders also include hyperkinetic dysfunction, with Huntington’s disease (HD) as a representative. Clinically, the disease is characterized by chorea, motor dysfunction, dystonia, cognitive impairment, mental disturbances (anxiety, depressive symptoms), and weight loss. At the molecular level, GABA-ergic neurons in the striatum are mostly affected, leading to an increased striatal DA level.

The rationale behind testing Que as a potential anti-HD agent is its neuroprotective activity. A currently used experimental HD model involves the administration of 3-nitropropionic acid (3-NP), which dysregulates mitochondrial metabolism, lowers the cellular ATP level, and increases oxidative stress, which eventually leads to nerve cell death. In a study conducted by Sandhir et al. [88] on female Wistar rats, Que administered orally at the dose of 25 mg/kg for 21 days (for 17 out of these 21 days concomitantly with 3-NP) mitigated motor deficits, assessed in narrow beam walk test and by footprint analysis. Furthermore, the molecular changes induced by 3-NP were reversed: mitochondrial suppression of respiratory chain complex, increased oxidative stress level, and a depletion in ATP concentration [88]. In addition, the 3-NP-induced alterations within the striatum were alleviated or absent. On the other hand, a study conducted by Chakraborty [89] failed to confirm the beneficial effect of Que on 3-NP-induced striatal neuronal lesion. Nevertheless, the conditions of the study varied a bit as Sandhir [88] used a subchronic dose of 3-NP, and in Chakraborty’s experiment [89], the dose of this neurotoxin was higher (20 mg/kg). Furthermore, the sex of the rats was male, and the duration of 3-NP and quercetin i.p. administration was only 4 days. Moreover, other symptoms such as weight loss, motor dysfunction, or anxiety were improved at the Que dose of 50 mg/kg. In another experiment, the combined protective potential of Que and lycopene (given p.o., at doses of 50 mg/kg and 25 mg/kg, respectively) on HD symptoms was studied in male Wistar rats, leading to an increase in the locomotor activity diminished by 3-NP, with no negative effect on the body weight [90]. Furthermore, the application of both compounds decreased behavioral deterioration (anxiogenic and depressive) often accompanying HD, in rats. These data seem inconsistent, and no equivocal conclusions can be drawn.

### 4.4. Other Neurodegenerative Diseases

Que was also investigated in single studies on models of some other diseases that are classified as neurodegenerative, such as amyotrophic lateral sclerosis (ALS) and multiple sclerosis (MS) (sometimes not classified as neurodegenerative for the alterations affect myelin sheaths, not whole neurons). The pathogenesis of both has an inflammatory background. Apart from the anti-inflammatory and antioxidant effect, Que was shown to reduce demyelination and stimulate remyelination in MS animal models [91]. In case of ALS, a deposition of aggregates of superoxide dismutase 1 (SOD1) is observed. Que, as well as other polyphenols, seems to be promising, as they can potentially suppress the elongation of fibrils, preventing SOD1 mutant fibrillation [92]. However, the existing experimental data are too scant to draw any conclusions as to their efficacy in these disorders.

## 5. Effects of Quercetin in Mental Disorders—Depression, Anxiety

### 5.1. Depressive Disorders

Depressive disorders are characterized by a group of symptoms, among which the following are usually highlighted: low, “depressive” mood, including sadness and feeling of emptiness, pessimistic thoughts, anhedonia, along with cognitive disturbances (e.g., difficulty in concentration, learning, making decisions); behavioral (e.g., anxiety); or neurovegetative symptoms (e.g., headache, stomachache, nausea, dizziness, sexual disorders). Brain regions affected by the changes causing symptoms of depression involve the prefrontal cortex (higher cognitive function as association, regulation of emotions), limbic system (reward system, feeling of pleasure), and basal ganglia with brain stem (automatic functions and reflexes), where the nuclei composed of neurons producing most neurotransmitters are located.

A detailed review on the activity of quercetin (Que) in animal models of depression, which covered studies up to 2020 has been recently released [7], so we would like to just briefly mention a few factors regarded as important in Que-exerted effect.

The most prominent hypothesis of pathogenesis of depression in the context of the search for novel therapeutic targets are: a disturbance in the levels of neurotransmitters (especially depletion of serotonin (5-HT), also noradrenaline and dopamine), dysregulation of HPA axis (chronic stress and chronically elevated serum concentration of cortisol is one of the factors inducing depressive-like disorders), and suppressing neurogenesis in the hippocampal dentate gyrus. Interestingly, the molecular effects exerted by quercetin are at some points close to those of known anti-depressants.

A recent study showed a positive influence on the expression of the hippocampal FoxG1/CREB/BDNF signaling pathway [93]. BDNF regulates phosphorylation and CREB activation, which in turn increases sympathetic neuron survival and is an important factor in short-term and long-term memory [88]. BDNF is involved in both depression and anxiety [94]. As stress is seen as a factor decreasing the expression of *Bdnf* genes, anti-depressants were shown to significantly elevate the BDNF level in brain regions such as the hippocampus or/and prefrontal cortex [94]. Trazodone, an anti-depressant with selective serotonin transporter inhibitor (SSRI) and 5-HT_2_ antagonist activity, reduced neuronal loss and stimulated neuronal survival rate and sertraline stimulated hippocampal neurogenesis [95]. Furthermore, it was reported that anti-inflammatory agents may possess anti-depressant potential [96] and some of known anti-depressants, e.g., fluoxetine, sertraline, or desipramine revealed anti-inflammatory activity [97,98].

In this context, neuroinflammation as a factor implicated in the pathogenesis of depression has been gaining more and more attention in recent years [14]. Thus, Que, which has a well-known anti-inflammatory activity, is considered as a potential anti-depressant. An inflammation-dependent decline in adult neurogenesis observed in depression is mainly linked to microglia-released cytokines such as IL-1β, IL-6, TNF-α, and INF-α [99]. Furthermore, oxidative stress is regarded as one of the main factors of neuronal damage, causing neurodegenerative as well as depressive disorders [100,101]. As was revealed in several experiments in animal models of depression, quercetin exerted antioxidant (reducing ROS, increasing SOD, GST, GSH activity) as well as anti-inflammatory activity (suppressing IL-1β, IL-6, TNF-α, COX-2, microglial activation) [102,103]. Furthermore, its regulating effect on the HPA axis was noted [104]. Some other studies reported Que’s interaction with 5-HT and NMDA receptors, which play essential role in the pathogenesis of depression [105]. Nevertheless, these data need confirmation in more in-depth research.

Anjaneyulu et al. [106] employed STZ-induced diabetic mice to assess the anti-depressive potential of Que. Fluoxetine and imipramine, reference drugs belonging to SSRI and tricyclic anti-depressant, respectively, exerted anti-depressant activity at the doses of 5 and 15 mg/kg (i.p.), respectively, whereas Que showed a dose-dependent effect but at significantly higher doses: 50 and 100 mg/kg (i.p.). In a recent study on a different model, i.e., unpredictable mild chronic stress in mice, the effect of Que (at doses of 15 and 30 mg/kg. p.o.) was comparable to fluoxetine (15 mg/kg) in the tail suspension test [93]. Furthermore, the more hydrophilic quercetin 4′-*O*-glucoside isolated from *Allium cepa* at a dose of 20 mg/kg was more potent than Que itself at the same dose, and its efficacy was comparable to fluoxetine (at 20 mg/kg). The inconsistency may result from different paradigms in the models used in the two experiments, as well as different schedules of administration and consequently different effects displayed by Que at the molecular level.

### 5.2. Anxiety Disorders

The main anxiety or fear-related disorders include: generalized anxiety disorder (GAD), panic disorder (PD), social anxiety disorder (SAD), and post-traumatic stress disorder (PTSD). Obsessive-compulsive disorders (OCD) are sometimes subclassified as anxiety disorders, as well. These disruptions are characterized by increased arousal and fear. It is agreed that γ-aminobutyric acid (GABA) transmission is disrupted in anxiety disturbances, wherein GABA is the main inhibitory CNS neurotransmitter. 5-HT and NA systems are also involved in dysregulation in physiological arousal and implicated negative emotions. Furthermore, one of the major factors regulating anxiety is the hypothalamus–pituitary–adrenal (HPA) axis, with the role of responding to stress.

In a couple of studies, the anxiolytic activity of Que was confirmed in vivo. The key data are summarized in Table 2. The most commonly utilized test to assess anxiolytic potential in rodents (mice, rats) was elevated plus-maze in different models of fear [53,67,90,102,104,107,108]. The effect of Que was significant, but in 5 out of 8 tests, the dose used was 50, 60, or even 100 mg/kg. The three other studies showed higher efficacy—lower doses exerted significant anxiolytic response (Table 2). Interestingly, two groups of researchers revealed that not all doses of quercetin were active, and the dose–response curve was bell- or U-shaped [108,109]. The same observation was made in the case of another anxiolytic, buspirone. This may be a result of the stimulation of autoreceptors which inhibit the postsynaptic response in a negative feedback loop. Only in three experiments were the standard drugs used as a reference—in two of these anxiolytics, diazepam or buspirone, their effective doses were much lower as compared to Que. In the case of one study, ibuprofen, which is a non-steroidal anti-inflammatory drug, was used, however, with no dose given. Lastly, one study used a marble-burying test (MBT) in mice paradigm to check anti-OCD potential [110]. Nevertheless, the effect of Que was reported only on the 12th day of the experiment, but not on the 11th, in a two-zone-modified version of MBT (the modification covers using only half of the space in the cage to arrange the marbles). The possible mechanisms which were postulated are mainly based on antioxidant and anti-inflammatory alterations that Que exerted. The results of one study suggested an involvement of GABA-ergic. Kosari-Nasab et al. [104] proved the engagement of Que in HPA axis functioning.

## 6. Therapeutic Potential of Quercetin in Brain Cancer

### 6.1. Cytotoxicity and Mechanisms

Brain cancers are characterized by rapid development, high drug resistance, and poor prognosis. Thus, there is a constant need to search for any additional agents that could be used in combination with chemotherapy and radiotherapy and improve the prognosis of the patients. Quercetin (Que) has been widely studied against many types of cancer cells, and its cytotoxic effect towards a number of murine and human brain cancer cell lines was also studied. Gliomas and glioblastomas, differing in their metastatic potential and/or drug resistance, were the most frequent cell lines used, with single papers describing the effect on astrocytoma [111,112,113] or neuroblastoma [114] cells. One study, by Lagerweij et al. [115], was focused on medulloblastoma cell lines. The reviewed papers, with few exceptions [116,117], usually did not include normal cells in the study, which makes it difficult to compare the safety and selectivity of the compound. The described cytotoxic effects of Que involved a number of different mechanisms, including apoptosis stimulation, often in combination with autophagy, inhibitory effect on cell migration, or angiogenesis, and the summary of the most important effects is presented in Table 3. Moreover, in a recent study, a molecular docking approach was described to verify Que multi-targeting potential against selected glioma targets, namely, epidermal growth factor receptor (EGFR), ephrin type-A receptor 2 (EphA2), nicotinamide phosphoribosyltransferase (NMRPTase), and plasminogen activator inhibitor-1 (PAI-1). Que interacted strongly via hydrogen bonding with important active sites of EphA2 and PAI-1, and the complexes were stable. The authors conclude that Que can provide a scaffold for designing novel anti-glioma therapeutic agents [118]. Another interesting strategy for combating glioma was presented by da Silva et al. [119], who focused on the modulation of the microglial inflammatory profile by Que. The treatment resulted in the increased expression of mRNA for IL-1β, IL-6, and IL-18 and decreased expression of mRNA for nitric oxide synthase 2 (NOS2) and prostaglandin-endoperoxide synthase 2 (PTGS2), arginase, and transforming growth factor beta (TGF-β), as well as insulin-like growth factor (IGF) in microglial cells, which suggests that Que may be considered as an adjuvant in the treatment of gliomas.

### 6.2. Synergism

A number of studies focused on evaluation of Que efficacy in combination with other approved drugs or other substances to verify the synergistic potential of the compound. Al-Hasawi et al. [111] reported that combined exposure to Que and cadmium (200 µM and 16 µM, respectively) reduced viability of astrocytoma 1321N1 cells to 6.9%, in comparison to the incubation with each of the substances alone. The effect was significantly increased with longer incubation time (48 h). The synergistic effect of Que and temozolomide on astrocytoma MOGGCCM cells was noted by Jakubowicz-Gil et al. [112]. At a low (5 µM) drug concentration, Que potentiated a pro-autophagic effect of temozolomide, while at higher drug concentration (30 µM), autophagy switched to apoptosis. The same authors observed synergistic effect of Que and sorafenib, manifested as the enhancement in proapoptotic properties of the drug on T98G glioma cells [113]. The synergy of Que and temozolomide (30 and 100 μmol/L, respectively) was also described by Sang et al. [130] on U251 and U87 glioma cells, with a significant decrease in cell viability, when compared to the substances alone. Moreover, neither temozolomide nor Que affected caspase-3 activity and cell apoptosis, while their co-treatment resulted in an increased caspase-3 level and apoptosis stimulation. Pozsgai et al. [131] compared the efficacy of treatment with irradiation, temozolomide, and Que, alone or in combinations, on DBTRG-05 and U-251 glioblastoma cells. The combined effect of irradiation and Que on cell viability and colony formation was comparable to the efficiency of the combination of irradiation and temozolomide. Moreover, Que combined with irradiation significantly activated apoptosis in the tested cells, while the combination with temozolomide and irradiation promoted necrosis. Que was also combined with chloroquine, an antimalarial agent, in the treatment of T98G, U251MG, and U373MG glioma cells. The combination of both substances caused autophagy in glioma cells, observed as the excessive expansion of autolysosomes and lysosomes, leading to cell death. Importantly, no toxic effect was noted for normal astrocytes [117]. In a study by Taylor et al. [132], Que was co-administered with sodium butyrate (25 μM and 1 mM, respectively) to rat C6 and human T98G glioblastoma cells, which resulted in the increase in apoptosis in the cells. An interesting idea of Que use in glioma U87 and T98G treatment was described by Tsiailanis et al. [133], who synthesized a novel and stable quercetin–losartan hybrid. The hybrid compound inhibited ROS and revealed antioxidant capacity but also lead to glioma cell cycle arrest and significantly decreased cell proliferation and angiogenesis in comparison to the parent components alone or their simple combination.

### 6.3. Novel Drug Delivery Systems

Some authors described the novel drug delivery systems for Que as a strategy for combating glioma. One of the most interesting studies involved the use of human platelets as drug delivery carriers loaded with Que in the U373-MG cell line. Platelets have an open canalicular system that allows the uptake of molecules in their cytoplasm. The authors obtained a three-fold enhancement of Que solubility, followed by the enhancement in cytotoxic effect (cell viability 14.52% after 48 h) after the use of this delivery platform for Que [134]. Other studies described the enhancement in cytotoxicity to rat C6 glioma cells after the use of nanoparticles loaded with Que [46,135] or induction of necrosis in the cells treated with Que nanoliposomes [136]. In two in vivo studies, nanoparticles with Que inhibited tumor progression in U97 glioma-bearing mice [137], while in another study, freeze-dried nanomicelles with Que administered to glioma-bearing mice accumulated in brain tissues and increased the survival of the animals [138].

Barbarisi et al. [139] proposed a nanohydrogel of hyaluronic acid loaded with Que alone or in combination with temozolomide to enhance the efficacy of the latter. The nanohydrogel loaded with Que targeted CD44 receptor on glioblastoma cells, thus improving the cytotoxic effect but also significantly reducing the production of pro-inflammatory mediators (IL-8, IL-6, and VEGF). A similar combination of Que and temozolomide, prepared in the form of nanoliposomes, was highly taken up by the U87 glioma cells, while, for both individual substances, the effect was only minimal. Importantly, the effect was also observed in temozolomide-resistant U87 cells in vitro, while in vivo experiments revealed a significant accumulation of the quercetin-temozolomide nanoparticles in rat brain, not observed for the individual compounds [140].

The cytotoxic potential of quercetin was also examined in vivo, but limited studies have been published within the timeframe of this review. In the study of Bi et al. [125], Que was administered i.v. to glioma C6-implanted rats (*n* = 24) alone (100 mg/kg) or in combination with chloroquine (100 mg/kg + 20 mg/kg) for two weeks. The enhanced antitumor effect was noted for the group that received the combination of both substances, as shown by the smallest tumor volumes and the smallest decreases in body weights, when compared to the groups receiving each of the substances alone or the control, untreated group. The survival time for the animals treated with the combined therapy was almost doubled, in comparison to other groups [125].

An interesting study was described by Lagerweij et al. [115], who applied Que together with irradiation during the medulloblastoma treatment. Que (100 mg/kg) was administered to mice i.p. 30 and 60 min before or after irradiation and 0 h and 24 h after irradiation, while the control group received the same volume of DMSO dissolved in PBS. The combination of quercetin and irradiation resulted in the increased survival of the animals when compared to the control group (median survival time of 32 and 12–17 days, respectively). The authors concluded that Que may be used as a radiosensitizer for medulloblastoma cells.

## 7. Clinical Studies on Quercetin in CNS Diseases

### 7.1. Case Study—Glioblastoma

Interestingly, apart from the above-mentioned studies on mice or rats, one human case study was also published. A patient with advanced multifocal and rapidly progressing glioblastoma multiforme was treated with standard radio- and chemotherapy (temozolomide), combined with intravenously administered Que at a single dose of 500 mg. Leading-edge gamma knife was included in the treatment protocol to improve the penetration of Que through the blood–brain barrier. The patient experienced improved quality of life and response, compared to historical data, including the ability to walk, requirement for steroids, and balance and coordination. The authors suggest that the inhibitory effect of Que on glioblastoma cells migration ability, described in a number of in vitro studies, is crucial for the decrease in disease progression [141].

Despite the promising results of the in vitro studies, the perspective for the use of Que in brain cancer therapy is still far-fetched. Many further studies are needed, including animal and human trials, to clarify its effectiveness. One of the most interesting and/or promising directions for future studies is the sensitizing potential of this compound, when combined with other therapies. More effort should be also made towards the development of novel delivery platforms for Que.

### 7.2. Clinical Study—Alzheimer’s Disease

Cognitive-enhancing activity seems to be the most promising with regard to Que future application. Interestingly, beneficial results of a vast number of molecular and preclinical studies encouraged researchers to perform clinical trials, especially since the compound is generally recognized as safe (GRAS). Based on an earlier study from 2010 [142] where 2000 mg of Que given immediately before the vigilance task induced a trend of increased effectiveness in the task (not statistically significant), in 2012, Broman-Bulks et al. [143] designed a study to check how a long-term ingestion of Que at doses usually recommended in supplementation would affect cognitive performance. A group of 941 participants received Que supplementation (500 or 1000 mg vs. placebo) daily for 12 weeks, and the effect on different aspects of neurocognitive functioning (memory, attention, psychomotor speed, reaction time, cognitive flexibility) was assessed. As a result, the CNS Vital Signs test battery, utilized to assess the performance of the participants before and after 12-week supplementation, exhibited no statistical effect on increased neurocognitive functioning. The authors concluded that the ergogenic effect of Que in humans is below the level expected based on the studies in mice.

Noteworthy, Que has reached phase II of the Clinical Trial to Evaluate the Safety and Feasibility of Senolytic Therapy in Alzheimer’s Disease. A rationale for the study were the results from an experiment performed in a mice model of AD, showing that the combinations of the drugs used in the trial protect neurons from dying. Moreover, Que and dasatinib have been recognized as senolytics, substances selectively eliminating senescent cells, contributing to many age-related or age-predisposed diseases [144]. They exerted more significant effects in vitro when given in combination than either alone. Participants enrolled for the clinical study are older patients with mild cognitive impairments or early stage-AD (tau-positive). A daily oral dose of 1000 mg of Que (Thorne Research^®^, which is in a form of phytosomes, a formulation with increased bioavailability) is given in 4 divided doses, along with a capsule of 100 mg of dasatinib. The combination of the drugs is given for 2 consecutive days, then a 13-day interval with no drug follows, and another 2 days of drugs application, and so on until 6 cycles of administration are finished. Preceding that trial, an initial study with the acronym SToMP-AD (Senolytic Therapy to Modulate Progression of Alzheimer’s Disease) was conducted to check if the drugs penetrated the brain by analysis of the cerebrospinal fluid [145]. Quercetin’s concentration in the blood as well AD-related markers were measured. Nevertheless, the results of this study have not been published as of yet.

## 8. Discussion and Conclusions

**Figure 4:**
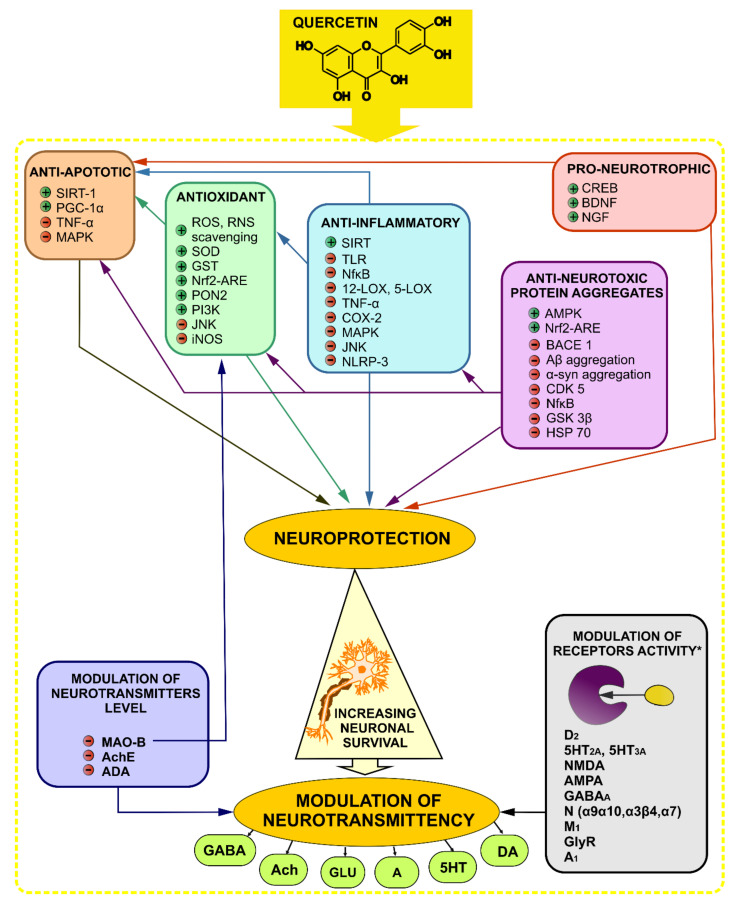
Mechanism of action of quercetin in mental and neurodegenerative disorders with main postulated molecular targets. + indicates a stimulation or an activation, − indicates an inhibition; Neuroprotective and modulatory neurotransmittency effects are responsible for Que activity in neurodegenerative and mental disorders. Neuroprotection is exerted by anti-apoptotic, antioxidant, anti-inflammatory, anti-neurotoxic protein aggregates, and pro-neurotrophic (increasing the release of neurotrophic factors) effects. Furthermore, this neuroprotective effect may positively influence on neurotransmission between neurons. Que may also modulate the level of neurotransmitters by inhibiting their enzymatic degradation. Moreover, in some research, the modulatory effect on receptors activity was shown for Que, although the data are inconsistent (*). 5-HT serotonin/serotonin receptor, A adenosine, A_1_ adenosine receptor 1, Aβ β-amyloid, Ach acetylcholine, AchE acetylcholinesterase, ADA adenosine deaminase, α-syn α-synuclein, AMPA α-amino-3-hydroxy-5-methyl-4-isoxazolepropionic acid receptor, AMPK AMP activated protein kinase, BACE1 β-secretase 1, BDNF brain-derived neurotrophic factors, CDK5 cyclin-dependent kinase 5, CREB cyclic-AMP-response element-binding protein, COX cyclooxygenase, DA dopamine, D dopaminergic receptor, GLU glutamate, GlyR glycine receptor, GSK3β glycogen synthase kinase 3 β, GSH glutathione, GST glutathione transferase, HSP70 heat shock protein 70, IL interleukin, iNOS inducible nitric oxide synthase, JNK c-Jun N-terminal kinase, LOX lipoxygenase, M muscarinic acetylcholine receptor, MAO-B monoamine oxidase-B, MAPK mitogen-activated protein kinases, N nicotinic acetylcholine receptor, NGF nerve growth factor, NMDA N-methyl-d-aspartate receptor, NfκB nuclear-kappa B factor, Nrf nuclear factor-like 2, NRLP-3 NLR Family Pyrin Domain Containing 3, PGC-1α peroxisome proliferator-activated receptor-gamma coactivator, PI3K phosphoinositide 3-kinases, PON2 paraoxonase-2, RNS reactive nitrogen species, ROS reactive oxygen species, SIRT sirtuin, TLR toll-like receptors, SOD superoxide dismutase, TNF-α tumor necrosis factor-α.

Quercetin (Que) is a safe, health-protective, and common food ingredient that is easy to isolate either in a pure or glycosylated form. Both are generally characterized by low bioavailability; nevertheless, as regards CNS diseases, the activity of pure Que has been studied most often, and there have been many novel formulations investigated to better deliver Que to its site of action. Que’s activity has been widely studied, and many pathways that contribute to its mode of action have been reported. The most relevant and prominent molecular and cellular targets in terms of therapeutic application in neurodegenerative and mental disorders are shown in Figure 4. However, it should be remembered that up till now Que is used only as a diet supplement supporting health and not as a drug aiming to cure a particular illness. Although Que seems to be promising in alleviating different symptoms of diseases as it is generally a highly reactive structure and subsequently its biological activity is pronounced, clinical studies are essential to prove its efficacy in the proposed dosage schedule and period of administration. Reviewing the available data on Que may give ideas regarding its potential application, showing the directions that when followed, and may fill the gaps in information needed to assess its therapeutic utility.

An analysis of the available literature on the effects of Que in animal models (preclinical studies) allows us to conclude that active doses of Que were moderate (10–30 mg/kg) or relatively high (up to even 70–100 mg/kg), indicating not very strong or even weak effect exerted by the compound [7,58,82,83]. The preclinical and clinical effectiveness of Que is limited due to its low water solubility, intensive first-pass liver metabolism, and weak blood–brain barrier permeability, which all result in low bioavailability in situ. Many researchers recognize these features as the reason for weak in vivo effectiveness despite undeniable activity. Although, in some recent studies, administration of only high doses of pure Que revealed remarkable effect, the exploration of novel drug formulations gives much hope as Que is effective at much lower doses (see Table 1). Administration of Que in novel drug formulations leads to its much higher concentrations in the brain (Table 1). One in vivo study utilized plasma exosomes loaded with quercetin [37]. Quercetin alone, naïve exosomes, and a complex of Que in exosomes were injected i.p. daily for 7 consecutive days into C57BL/6 mice with okadaic-acid-induced AD. Results have shown that mice administered with Que in exosome carriers performed significantly better in Morris water maze testing spatial learning and memory than those which received quercetin alone. Furthermore, the apoptotic rate in neuronal cells was lower in the exosome-quercetin group. These results are accordance with data on Que concentration in the brain, which was very differentiated between the study groups. Additionally, no toxicity of the exosome–quercetin complex towards different organs was noted. In another study, exosome–quercetin complexes inhibited the activity of CDK5 and NFTs formation more strongly than the flavonoid alone [48]. A murine Aluminum-induced model of AD, characterized by SPs and NFTs formation, astrogliosis, and impaired neurogenesis in hippocampal gyrus dentate corresponding to the alterations in AD, was utilized in another study investigating the activity of Que nanoparticles (QNPs) [146]. The compound given at 30 mg/kg significantly reduced neuron degeneration with regenerative effect and other mentioned changes observed in the AD model. Although this study did not investigate Que alone, it provided some very interesting data on the comparison between prophylactic and treatment intervention, and interestingly, the prophylactic effect was remarkably higher.

Needless to say, the more similar the study conditions are to those of the prospective drug administration, the more conclusive its results. From that point of view, the in vivo investigations with oral route of administration reflect more faithfully the conditions of Que’s application in humans. Importantly, most of the analyzed studies involved Que applicated per os.

The main weakness of most of the published reports was the lack of a positive control. This hinders drawing unequivocal conclusions regarding quercetin’s efficacy, as it is difficult to assess whether the observed effect is comparable, lower, or maybe higher than that of an approved reference. One of the reasons behind these experimental inaccuracies may be a limited number of approved drugs that are used to treat AD or other types of dementia and their controversial efficacy. However, no control was used in tests assessing activity in PD animal models, although there are several drugs available which increase CNS dopaminergic activity. Nevertheless, in case of HD, no efficacious pharmacotherapy is available. Yet, it is surprising that the effect of Que was compared to a reference anti-depressant or anxiolytic drug only in a few works, even though there are quite a lot of drugs belonging to these groups that can be used as comparators.

Furthermore, instead of study protocols using different methods confirming the effect, solely one is commonly applied in experiments. That was in case of anxiolytic activity investigations of Que discussed in this review. It is agreed that different animal models have their limitations and explore different factors controlling behavior/kinds of fear (elevation, open space, stimuli). Consequently, to confirm different aspects of fear behavior different models should be employed. Nevertheless, in most reports, the elevated-plus maze test, triggering fear linked to open space and elevation, is a sole method used to assess in vivo anxiolytic activity, whereas there are other tests that can be utilized to widen the knowledge on the different components of the effect exerted by the tested substance. These are light/dark box in mice, four-plate test in mice, or conflict Vogel test in rats. The two latter induce fear by an electric stimulus.

Another important factor when comparing results from different studies is the species used in in vivo tests. A potential activity revealed by a given substance in experiments on more than one species (most commonly mice and rats) makes the results more reliable. That is why it is so important to accumulate the results of studies utilizing different models of the disease and animal species.

All in all, taking into consideration the described effects of Que in neurodegenerative disorders, it seems obvious that the most promising direction of further pharmacological studies on this compound is either anti-dementia or pro-cognitive action. Data on Que’s efficacy in PD models are inconsistent and demand more in-depth analysis. In case of HD, apart from several experiments, a potential use of Que is not being currently explored as, at this point, the results seem not very promising or convincing. However, there is still much to be explored in terms of HD as no effective treatment option is currently available.

Furthermore, in terms of mental disorders the anti-depressant potential of Que in animal models is undeniable. Still, as was mentioned, not much is known on the comparison of its efficacy with currently used anti-depressants. What is more, in the light of current knowledge regarding Que bioavailability, which is the limiting factor of its efficacy, the studies using quercetin-loaded novel drug delivery systems are missing. Furthermore, to the best of our knowledge, no clinical observations assessing the ability of Que to exert an effect in chronic supplementation in patients with depression were made.

Another promising direction of research seems to be anxiolytic activity of Que. Especially in view of the fact that anxiety disruption can be a co-symptom of depression or many other diseases, not only of the CNS. Yet, as mainly higher doses were active in the described studies, a battery of experiments including novel formulation should be designed to assess the efficacy of Que in conditions of enhanced bioavailability.

Que, either as a free compound or in a glycosidic form, or as a part of a plant extract (*Sophora japonica* is a common example, as it is a good source of rutin), is a common ingredient of many dietary supplements. The compound is recommended in a daily dose ranging from 200 to 1000 mg, whereas amounts from 10 to 25 mg per portion are usually found when it is used as food additive. The most effective dose of Que in animal models was most frequently up from 25 mg/kg, but there are studies where the compound was active at lower (5 mg/kg) or only higher doses (50, 100 mg/kg) [21]. Assuming that an adult woman weighs about 60 kg and a man ca. 70 kg, a dose of 25 mg/kg, would amount to 1500 and ca. 1900 mg/per day, respectively. This is about twice as much as the dose recommended in dietary supplements, but we need to remember that medicinal products are supposed to treat illnesses and not only support the organism as in the case of supplements.

Moreover, what we can learn from the results of animal studies and the proposed mechanism of action, assuming the changes in gene expression and long-term effects within CNS, is that a positive impact on health can be achieved by the chronic administration of Que.

## 9. Summary

Undoubtfully, Que is a biologically active agent with apparent impact on the CNS, which may be of future use in the treatment of CNS disorders, presumably as an adjuvant, not in monotherapy. The clinical studies in terms of cognitive improvements or the described case study in brain cancer prove its potential. Recently, a combination of Que with dasatinib is subject to wide research in terms of senolytic activity. Furthermore, the development in technology of novel drug delivery systems gives hope to develop Que drug formulations with better BBB penetration and a more powerful effect already at lower doses.

## Figures and Tables

**Table 1:** Novel formulations of quercetin and their effects in in vitro and in vivo models.

Formulations	Animal Model/Doses/Route of Administration	Conclusions	Ref.
Quercetin-loaded cationic nanostructured lipid carriers (QR-CNLC)	In vivo study Healthy male C57BL/6J mice; oral administration of: (a) quercetin suspended in 0.5%, *w*/*v* CMC-Na aqueous solution (b) QR-CNLC at a dose of 25 mg/kg bwt	QR-CNLC failed to accumulate higher quercetin in brain tissue than quercetin suspension	[34]
Quercetin conjugated with superparamagnetic iron oxide nanoparticles (QT-SPION)	In vivo study: Healthy Wistar male rats orally fed by gavage with: (a) quercetin solution (b) QT-SPION at a dose of 50 and 100 mg/kg/d for 7 days	QT-SPION ↑ the bioavailability of quercetin in the brain	[33]
Quercetin-loaded poly(n-butylcyano acrylate) nanoparticles (QT-PBCA NPs); QT-PBCA NPs coated with polysorbate-80 (P-80) on their surfaces (QT-PBCA + P-80)	In vivo study Wistar male albino rats; administration of: (a) quercetin (b) QT-PBCA NPs (c) QT-PBCA + P-80 at a dose of 50 mg/kg bwt via oral feeding cannula	QT-PBCA NPs ↑ the quercetin bioavailability by 2.38-fold QT-PBCA + P-80 ↑ the quercetin bioavailability by 4.93-fold	[35]
Quercetin-loaded nano lipidic carriers employing phospholipids and tocopherol acetate (NLCs); Quercetin-loaded solid lipid nanoparticles (SLNs)	In vivo study Wistar male rats; oral gavage of: (a) quercetin (b) NLCs (c) SLNs in equivalent quercetin doses of 50 mg/kg	SLNs ↑ the brain delivery of quercetin by 3.2 times ↑ the bioavailability of quercetin by 3.5-fold NLCs ↑ the brain delivery of quercetin by 5.6 times ↑ the bioavailability of quercetin by 5.4-fold ↑ neuroprotective activity	[36]
Plasma exosomes loaded with quercetin (Exo-Que)	In vivo study Okadaic acid-induced model of tauopathy and cognitive deficiency; AD mice; peritoneal injection of (a) quercetin (b) Exo-Que	Exo-Que ↑ quercetin concentration by 2.5-fold in cerebrum and 1.5-fold in cerebellum ↑ effect in MWM test	[37]
Quercetin-modified sulfur nanoparticles (Qc@SNPs) embedded in microbubbles (MB) (Qc@SNPs-MB)	In vivo study Fluorescence real-time imaging concentration of ruthenium-labelled NPs: 1 mg/kg MWM test in AD mice, NPs given i.v., 2 days/week for 5 weeks, concentration of both NPs: 5 mg/kg Concentration of NPs = 10 μg/mL	Qc@SNPs ↑ the brain targeting and bioavailability of quercetin ↓ ER stress in nerve cells	[38]
Quercetin-loaded zein-based nanofibers developed using electrospinning technique	In vivo study Streptozotocin induced diabetic rat model; adult Wistar male rats; animals exposed to crush injury and subjected to zein-based nanofibers loaded with quercetin at concentrations of: (a) 5% (b) 10% (c) 15% for 21 days	Different concentrations of quercetin can be loaded into nanofibers without differences in their diameters. The release of quercetin from tested nanofibers was >60% within first 6 h, obtained a maximum within 24 h, and lasted at least 72 h. Quercetin-loaded, zein-based nanofibers ↑ the effect of quercetin in neuropathic injury in rats	[39]
Quercetin conjugated superparamagnetic iron oxide nanoparticles (QCSPIONs)	In vivo study Sreptozotocin induced diabetic rat model; adult Wistar male rats orally fed by gavage with: (a) SPIONs (b) quercetin (c) QCSPIONs at a dose of 25 mg/kg for a period of 35 consecutive days	QCSPIONs ↑ SOD1 and CAT expression levels ↑ learning and memory in diabetic rats ↑ Nrf2 and antioxidant genes expression level by miR-27a regulation	[40]
**Formulations**	**Assay**	**Conclusions**	**Ref.**
Quercetin-loaded nanoemulsion prepared using spontaneous emulsification technique (QUR-loaded NE)	In vitro study Dialysis membrane method; isolated sheep nasal mucosa; QUR-loaded NE	QUR-loaded NE for intranasal administration seems to be a promising delivery system for anticancer agents to achieve CNS targets	[41]
Fast-dissolving core-shell composite microparticles of quercetin fabricated using coaxial electrospraying	In vitro study Permeation study performer using a RYJ-6A Diffusion Test Apparatus Quercetin contents: (a) M2 (262 mg) (b) M3 (187 mg) (c) M5 (120 mg) (d) quercetin (20 mg)	Fast-dissolving core-shell composite microparticles of quercetin ↑ the dissolution and permeation rates of quercetin	[42]
Quercetin-loaded β-CD dodecylcarbonate nanoparticles	In vitro study Dialysis bag method; SH-SY5Y cells; quercetin-loaded β-CD dodecylcarbonate nanoparticles	Quercetin-loaded nanoparticles ↑ BBB permeation, bioavailability, and access to target cells ↑ neuroprotective efficacy	[43]
Quercetin-modified polysorbate 80 (P-80)-coated AuPd nanoparticles (Qu@P-80@AuPd)	In vitro study Transwell co-culture system; bEnd.3 cells; SH-SY5Y cells to co-culture; flow cytometric method to measure transport efficiency through BBB of: (a) Concave cubic Qu@AuPd at a dose of 10 μg/mL (b) Concave cubic Qu@P-80@AuPd at a dose of 10 μg/mL	Concave cubic Qu@P-80@AuPd ↑ BBB permeability and good biocompatibility in Transwell assay, MTT, and apoptosis Concave cubic Qu@P-80@AuPd could have a potential in AD treatment as an autophagy inducer	[44]
Quercetin-loaded poly(n-butylcyanoacrylate) (PBCA) nanoparticles (QT-PBCA NPs) Quercetin-loaded poly(n-butylcyanoacrylate) (PBCA) nanoparticles coated with polysorbate-80 (P-80) (QT-PBCA + P-80)	In vitro study Dialysis bag method; (a) quercetin (b) QT-PBCA NPs (c) QT-PBCA + P-80	QT-PBCA + P-80 ↑ oral bioavailability of quercetin ↑ the BBB penetration and CNS efficacy	[35]
Quercetin nanoparticles developed by pulsed laser ablation in water (Que NPs)	In vitro study Dialysis-based in vitro drug release assay; (a) Que NPs (b) quercetin powder in PBS	Que NPs ↑ bioavailability of quercetin ↑ efficacy of quercetin as a result of prolonged residence time in systemic circulation	[45]
PEG2000-DPSE-coated quercetin nanoparticles (PEG2000-DPSE-QUE-NPS)	In vitro study Annexin V-FITC K; Mitochondrial Membrane Potential Assay Kit; glioma C6 cells; PEG2000-DPSE-QUE-NPS	PEG2000-DPSE-QUE-NPS ↑ solubility of quercetin ↑ efficacy of quercetin in inhibiting glioma C6 cells through induced apoptosis and necrosis.	[46]
Quercetin-loaded nanolipidic carriers employing phospholipids and tocopherol acetate (NLCs); Quercetin-loaded solid lipid nanoparticles (SLNs)	In vitro study DPPH antioxidant assay; Caco-2 cellular permeability study; (a) quercetin (b) NLCs (c) SLNs (d) ascorbic acid	NLCs and SLNs ↑ quercetin intestinal permeability ↑ antioxidant effect	[36]

Abbreviations: Caco-2—human colon adenocarcinoma cell line; CMC-Na—sodium carboxymethyl cellulose; MWM test—the Morris Water Maze test; PBS—phosphate-buffered saline; SH-SY5Y—human neuroblastoma cell line; Ref.—reference; ↑—an increase in the effect; ↓—a decrease in the effect.

**Table 2:** Anxiolytic activity of quercetin—in vivo studies.

In Vivo Study	Treatments	Results	Ref.
EPM in pregnant female Wistar rats acutely stressed by a predator (a cat)	quercetin: 50 mg/kg p.o., for 6 days (from 14th to the 19th day of gestation)	Anxiolytic effect of quercetin. A significant decrease in the elevated by the stressor corticosterone level, alleviation of oxidative stress (reduced GSH, increased GST).	[93]
EPM in 2.5 mg/kg CD-intoxicated male Wistar rats	quercetin: 5, 25, 50 mg/kg p.o., administered 5 days a week for 45 days	Inhibiting anxiogenic effect of Cd at all the doses.	[46]
EPM in male Wistar rats with 3-nitropropionic acid (3-NP)-induced Huntington’s disease	quercetin: 50 mg/kg p.o., lycopene: 25 mg/kg p.o.; given along with 3-NP for 14 days	Anxiolytic effect of quercetin given along with lycopene. The effect was not observable in case of a single substance, but it seemed the effect of lycopene was stronger than quercetin.	[94]
EPM in ICR mice	quercetin: 1.25, 2.5, 5, 10 mg/kg p.o., 1 h before the test; buspirone: 2 mg/kg i.p.; 30 min before the test	Anxiolytic effect of 5 mg/kg quercetin (bell-shaped dose–response curve), comparable with 2 mg/kg buspirone, with no muscle relaxant effect or influence on locomotor activity. The effect was mediated by GABA-ergic system.	[95]
EPM in adriamycin (ADR)-injected male Wistar rats	quercetin: 60 mg/kg i.p.; 24, 5, and 1 h before the test session	Anxiolytic effect of quercetin. Oxidative stress level was alleviated (GSH maintained at high level; products of lipid peroxidation eliminated).	[97]
MBT in male albino mice	quercetin: 5 mg/kg, p.o., triethylene glycol (TEG): 5 mg/kg p.o.; once daily for 11 (standard, one-zone MBT) or 12 days (two-zones MBT)	Anti-obsessive-compulsive effect of quercetin but only in 12th day of experiment in 2-zone version of MBT.	[100]
EPM in STZ-induced diabetic male Wistar rats	quercetin: 5, 25, 50 mg/kg p.o.; for 40 days	Anxiolytic effect of quercetin, significant at all tested doses.	[27]
EPM, light-dark box, zero maze in mild traumatic brain injury (mTBI)-induced NMRI mice	quercetin: 50 mg/kg p.o., diazepam 3 mg/kg p.o.; once daily for 14 consecutive days (days 10–24 postinjury)	In all the tests, quercetin exerted significant anxiolytic effect, which was comparable to diazepam (although the dose was much higher). HPA axis was normalized by the drugs (ACTH and corticosterone level were decreased vs. mTBI group).	[96]
EPM in LPS-lateral ventricle-injected in male SD rats	quercetin: 50 and 100 mg/kg i.p., ibuprofen (the dose not showed) i.p.; once daily for 21 days after LPS injection	Anxiolytic effect of quercetin was dose-dependent but only at the dose of 100 mg/kg was a significant effect noted. Anxiety index was comparable low with ibuprofen. A reduction in inflammatory response was observed: a decrease in inflammatory markers: enzyme, COX-2, and cytokines, e.g., IL-1β, IL-6, NF-κB, in expression of inducible NOS and an increase in expression of BDNF.	[98]
tank tests in zebrafish (*Danio rerio*)	quercetin: 0.01, 0.1, 1, 10, 100, 1000 μg/L	At the lower doses anxiolytic effect, but the highest dose was angiogenic. The molecular mechanism involves alteration in inflammatory (an increase in antioxidant enzymes, e.g., SOD, a decrease in pro-inflammatory enzyme, COX-2, and cytokines, e.g., IL-1β, IL-6, IL-10, TNF-α), a suppression in an apoptotic response.	[99]

EPM—elevated plus-maze; MBT—marble-burying test.

**Table 3:** Molecular mechanism of cytotoxic activity of quercetin.

Biological Effect	Cellular Mechanism	References
apoptosis (intrinsic pathway)	cytochrome c release mitochondria membrane depolimerization caspase-3 and -9 PARP-1 cleavage MAPK cascade Bcl-2, survivin p53 NF-κB TNFα	[114,116,120,121,122,123]
cell cycle arrest	cyclin D1, D2 p21 CDK2	[124]
autophagy	Beclin-1 protein LC3 protein	[120,125,126,127]
inhibition of angiogenesis	capillary formation endothelial cells proliferation mTOR	[128]
inhibition of metastasis and migration	VEGF, MMP-2, MMP-9, fibronectin migration ability in wound healing and/or transwell assay	[123,124,126,127,128,129]

## Data Availability

Not applicable.

## Abbreviations

3-NP	3-nitropropionic acid
5-HT	serotonin/serotonin receptor
A	adenosine
A_1_	adenosine receptor 1
Aβ	β-amyloid
Ach	acetylcholine
AchE	acetylcholinesterase
AD	Alzheimer’s disease
ADA	adenosine deaminase
α-syn	α-synuclein
AMP	adenosine monophosphate
AMPA	α-amino-3-hydroxy-5-methyl-4-isoxazolepropionic acid receptor
AMPK	AMP activated protein kinase
APP	amyloid precursor protein
BACE1	β-secretase 1
Bcl	B-cell lymphoma
BDNF	brain-derived neurotrophic factors
CDK	cyclin-dependent kinase
CNS	central nervous system
COX	cyclooxygenase
CREB	cyclic-AMP-response element-binding protein
DA	dopamine
D	dopaminergic receptor
EGFR	epidermal growth factor receptor
EphA2	ephrin type-A receptor 2
GABA	γ-aminobutyric acid
GLU	glutamate
GlyR	glycine receptor
GSK3β	glycogen synthase kinase 3 β
GSH	glutathione
GST	glutathione transferase
HD	Huntington’s disease
HPA	hypothalamus-pituitary-adrenal
HSP70	heat shock protein 70
IL	interleukin
iNOS	inducible nitric oxide synthase
ISR	integrated stress response
JNK	c-Jun N-terminal kinase
LC3	microtubule-associated proteins 1A/1B light chain 3B
LOX	lipoxygenase
M	muscarinic acetylcholine receptor
MAO-B	monoamine oxidase-B
MAPK	mitogen-activated protein kinases
MMP	metalloproteinase
mTOR	mammalian target of rapamycin kinase
N	nicotinic acetylcholine receptor
NGF	nerve growth factor
NMDA	N-methyl-d-aspartate receptor
NMRPTase	nicotinamide phosphoribosyltransferase
NOS	nitric oxide synthase
NfκB	nuclear-kappa B factor
NFTs	neurofibrillary tangles
Nrf	nuclear factor-like 2
NRLP-3	NLR Family Pyrin Domain Containing 3
PAI-1	plasminogen activator inhibitor-1
PD	Parkinson’s disease
PGC-1α	peroxisome proliferator-activated receptor-gamma coactivator
PI3K	phosphoinositide 3-kinases
PON2	paraoxonase-2
Que	quercetin
RNS	reactive nitrogen species
ROS	reactive oxygen species
SIRT	sirtuin
SOD	superoxide dismutase
SPs	senile plaques
TLR	toll-like receptors
TNF-α	tumor necrosis factor-α
VEGF	vascular endothelial growth factor
